# Validation of the Clinical Frailty Scale for predicting 90-day mortality in hospitalised older adults screened as at risk of nearing the end of life in Queensland, Australia: a multisite observational study

**DOI:** 10.1136/bmjopen-2025-108419

**Published:** 2025-11-12

**Authors:** Robin Blythe, Nicole Marie White, Christine Brown, Ken Hillman, Adrian Gerard Barnett

**Affiliations:** 1 Programme in Health Services Research & Population Health, Duke-NUS Medical School, Singapore; 2 Australian Centre for Health Services Innovation and Centre for Healthcare Transformation, School of Public Health & Social Work, Faculty of Health, Queensland University of Technology, Kelvin Grove, Queensland, Australia; 3 The Simpson Centre for Health Services Research,Australian Institute of Health Innovation, University of New South Wales, Kensington, New South Wales, Australia; 4 University of New South Wales School of Clinical Medicine, Sydney, New South Wales, Australia; 5 Ingham Institute for Applied Medical Research, Liverpool, New South Wales, Australia

**Keywords:** Frailty, GERIATRIC MEDICINE, PALLIATIVE CARE, Sensitivity and Specificity

## Abstract

**Abstract:**

**Background:**

The Clinical Frailty Scale is an ordinal scale from 1 (very fit) to 9 (terminally ill) commonly used to assess frailty in older patients. It is simple for clinicians to apply and can help identify patients who may benefit from discussions around end-of-life care. We externally validated the Scale to assess its performance for predicting 90-day mortality in a cohort of admitted older patients who had screened positive for being at risk of nearing the end of life.

**Methods:**

We used data from a randomised controlled trial assessing a tailored feedback loop for reducing non-beneficial care. Our study included patients aged 75 and above admitted between May 2020 and June 2021 from 3 Australian hospitals. We assessed whether the Scale could be used in a frail population to identify patients who were at risk of short-term mortality. Predictive performance was assessed using the c-statistic, smoothed calibration curves and decision curves. Models were tested for coefficient stability.

**Results:**

Our dataset contained 4639 patients and 956 deaths within 90 days. The Clinical Frailty Scale had a c-statistic of 0.62 (95% CI 0.60 to 0.64) or 0.63 (95% CI 0.61 to 0.65) by adding age and transforming the Scale using a cubic spline. Risks were underestimated without a non-linear transformation as scores of 8 and 9 had a higher risk that diverged from a linear association. The net benefit of using the tool was greatest between 5 and 8 on the Scale.

**Conclusions:**

The utility of the Clinical Frailty Scale may be as a flag to encourage clinicians to become more comfortable with discussing ageing and death, rather than as a highly discriminating model to classify patients as high risk or low risk. Statistical uncertainty over mortality should not be a barrier to initiating end-of-life care discussions with frail older patients.

**STRENGTHS AND LIMITATIONS OF THIS STUDY**

- The Clinical Frailty Scale is very broadly used, and this paper deepens understanding of the tool in terms of its relationship with mortality.
- We show how the predictions made by the scale align with clinical expectations, including risk overestimation and underestimation, to inform clinical care.
- As this study used data from an end-of-life care intervention, patients were already considered to be at some level of baseline frailty, potentially limiting generalisability.
- Adjustments made to the scale to enhance performance detract from its ease of use and potential uptake by clinicians.

## Background

 For many older persons, hospitalisation can lead to the provision of unwanted or non-beneficial treatment. End-of-life care discussions between patients, their families and their care providers can be difficult to initiate, but can help align treatment with the patient’s goals.^1^ Non-beneficial treatment can be costly, unpleasant and inconsistent with people’s goals of care, making it important to recognise when frail older patients are nearing the end of life.^2^ However, determining which patients might benefit from these discussions is challenging, and clinicians may neither acknowledge nor discuss the end of life with colleagues or family members.^3^

Frailty and all-cause mortality are closely linked. Meta-analysis has identified that frailty scores are associated with higher risks of all-cause mortality.^4^ This association has been observed between the Clinical Frailty Scale and 30-day mortality, leading to conclusions that the tool is a predictive model capable of stratifying older emergency department (ED) patients by their risk of short-term mortality.^5^ The Clinical Frailty Scale combines clinical judgements about the patient, including self-sufficiency and capacity for activities of daily living, into a value from 1 (very fit) to 9 (terminally ill).^6^ Its ease of use, inter-rater reliability,^7^ evidence basis as a validated measure of frailty,^8^ and widespread application make the Clinical Frailty Scale a good candidate for predictive validation.

Prior research on the predictive value of the Clinical Frailty Scale has focused on its association with mortality, expressed as ORs or HRs. Ratios and other measures of association examine relative, rather than absolute, risks. They are therefore useful for understanding whether there is a relationship between a measure and an outcome at the population level, but of low value for assessing risk in individual patients.^9^ In individuals, ratios provide little information on the consistency of scores relative to the outcome, the probability of the outcome, and accordingly, whether a clinician needs to intervene based on the predicted score. In the case of the Clinical Frailty Scale, scores may be used to determine whether end-of-life care discussions should be initiated, and it is therefore important to understand its discriminating performance, the degree of individual risk overestimation or underestimation, and the expected benefit of using the tool for clinical decision-making.

In this study, we externally validate the Clinical Frailty Scale for the prediction of 90-day all-cause mortality in a population of older hospitalised adults.

## Methods

Prior validations of the Clinical Frailty Scale^10^ have focused on population-level associations between the scale and various outcomes relating to frailty, including mortality.^6^ We examined the prognostic value of the Clinical Frailty Scale as a clinical prediction model for all-cause 90-day mortality in an older, hospitalised population who may benefit from initiation of end-of-life care discussions. We aimed to thoroughly examine the scale, assessing its discriminating ability as well as:

- The calibration of the model, or the agreement between the number of deaths observed in the population and the number predicted by the model. This is often overlooked when prediction models are validated.^11^
- The net benefit of using the model for decision-making, or the rate of true positives minus false positives, weighted by the odds of the outcome. This provides useful information on clinical value.^12^
- How robust the model was to heterogeneity in the sample. This is important for understanding the expected generalisability of the model performance measures.^13^
- Whether the addition of age as a predictor improved the performance of the model in a frail population of older adults.

We prespecified an analysis plan by creating a dummy dataset with randomly generated values for patient age, Clinical Frailty Scale, admission date and date of death.^14^ We then used the dummy data to demonstrate preliminary results to the broader research team before repeating the analysis on the real data. All analyses were conducted in R (V.4.4.0).^15^ Adherence to best practices in external validation is reported in the Transparent Reporting of a multivariable prediction model for Individual Prognosis or Diagnosis checklist^16^ in the online supplemental file 1.

### Data collection

We used data from the Intervention for Appropriate Care and Treatment (InterACT) trial, registered with the Australian New Zealand Clinical Trial Registry (ACTRN12619000675123). InterACT tested whether a tailored feedback loop could reduce non-beneficial care for older patients admitted to hospital potentially nearing end of life.^17^ The main results of the trial have been published,^18 19^ and this analysis was in addition to the planned protocol. After the trial ended, patients’ details were linked with registered deaths, including deaths outside of hospital. Deaths up to 1 July 2021 were included. Patients who were still alive at the end of the trial were right-censored.

The InterACT trial was conducted in three large tertiary hospitals in Queensland, Australia between May 2020 and June 2021. Patients aged 75 and over who were admitted to hospital under the care of participating clinical teams were screened using two tools designed to identify patients towards the end of life. The two tools were: (1) The Criteria for Screening and Triaging to Appropriate aLternative Care (CriSTAL)^20^ and (2) Supportive and Palliative Care Indicators Tool.^21^ Patients were included in the trial if they were positive on either tool, which was 64% of patients screened.^18^ The Clinical Frailty Scale^22^ was a component of the CriSTAL tool and was generally attributed by clinical staff at each hospital on admission. Where the Clinical Frailty Scale was not readily available, criteria used in the scale, for example, activities of daily living, were gathered from admission notes.

**Figure 1:**
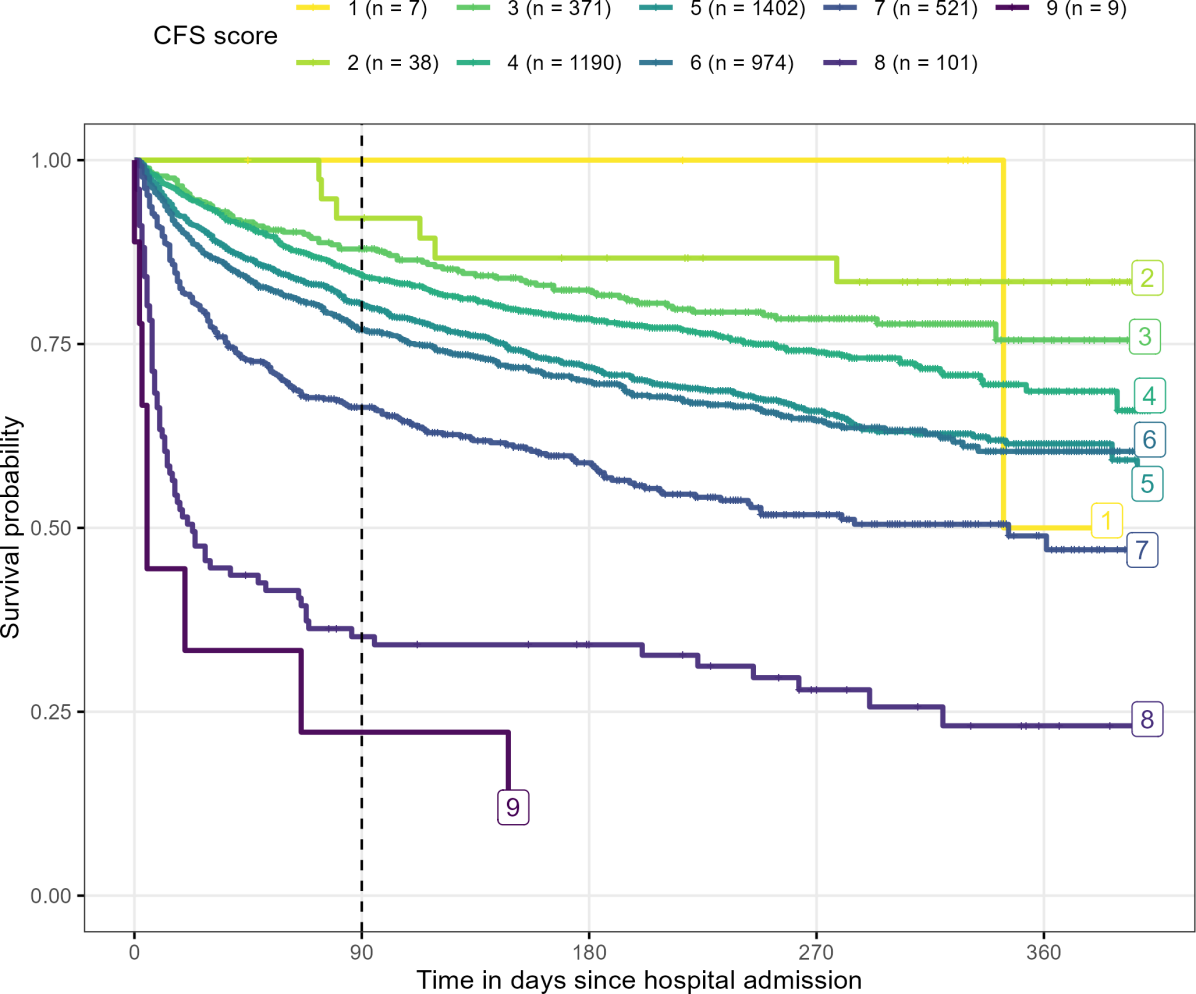
Survival curves by Clinical Frailty Scale (CFS) across all hospitals. Sample sizes for each CFS during screening are shown on the right. The notch on each line denotes right-censoring from patients still alive when data collection ended, while each downward movement denotes a death. The dashed vertical line at 90 days is the time at which patient survival was evaluated.

This population was deemed suitable for an external validation for several reasons. First, given the relationship between frailty and all-cause mortality, it is more likely that the 90-day mortality observed in this population was linked to frailty than in the general population of adults over 75. Second, older adults identified as potentially frail should be the target for interventions relating to end-of-life care, and the Clinical Frailty Scale is likely to be frequently used to differentiate between adults in this population. Finally, performance measures for any prediction tool should include its relationship to the outcome across the range of plausible values, and our sample displayed the full range of Clinical Frailty Scale scores (figure 1).

### Patient and public involvement

As this was a secondary analysis of deidentified data, it was not possible or feasible to include patients in the design, conduct or reporting of our research.

### Model specification

We selected death within 90 days as our outcome measure for two reasons. First, prior research has identified that when selecting a time frame for mortality outcomes, the choice should differentiate between mortality resulting from hospitalisation and from a more generalised state of frailty.^23^ Identifying patients who are likely to die over a longer time frame of 90 days is likely more relevant to frailty than their hospitalisation or any subsequent complications.^24^ Second, shorter time frames may be insufficient for patients to reconcile their prognosis with their care providers and families, which can lead to significant distress.^25^

Additive regression was used to singly impute missing data.^26^

In an exploratory analysis, we included the time from hospital admission to death (or right-censoring) and visually inspected the survival curves for each category of the Clinical Frailty Scale score using a Kaplan-Meier plot.

Previous studies have evaluated the Clinical Frailty Scale as a binary or ordinal value^10 27^; however, this potentially discards useful information across the range of possible scores and is inadequate for examining model calibration. To be able to estimate probabilities of death using the Clinical Frailty Scale, we first fitted a simple logistic regression model with Clinical Frailty Scale as a continuous variable (model 1):


$$logit\left(p_{90\;day\;mortality}\right)=\;\beta_{0}+\;\beta_{1}CFS.$$

To test whether age had any additional value to the Clinical Frailty Scale, we fitted a nested model with age (model 2):


$$logit\left(p_{90\;day\;mortality}\right)=\;\beta_{0}+\;\beta_{1}CFS+\;\beta_{2}Age\;on\;admission.$$

The addition of age was compared with the model with the Clinical Frailty Scale alone using the likelihood ratio χ² test. We then assessed whether age could be non-linear by applying a restricted cubic spline term (model 3),^28^ using the Wald test to assess linearity.

Our study required sufficient sample size to accurately estimate the coefficients of the Clinical Frailty Scale, β_1_, and age on admission, β_2_.^29^ We applied the methods of Riley *et al*,^30^ assuming our model required up to 5 df, a conservative c-statistic of 0.6 and an outcome prevalence of 20%. We comfortably exceeded the minimum sample size of 2260 patients and at least 452 deaths within 90 days. To assess variability in our sample, we also assessed model stability, repeating the entire model fitting process using bootstrapping with replacement.^31^ Predicted probabilities were assessed using calibration plots and mean average prediction error (online supplemental file 1).

### Model performance

Model discrimination demonstrates whether patients with a higher Clinical Frailty Scale are at higher risk of dying within 90 days than patients with a lower Clinical Frailty Scale. We measured discrimination using the c-statistic, also known as the area under the receiver operating characteristic curve (AUC).^32^ Calibration measures the relationship between observed and predicted mortality. If the Clinical Frailty Scale underestimates mortality risks, then fewer patients may receive potentially beneficial end-of-life care discussions. Conversely, if risks are overestimated, the Scale would flag too many patients and unnecessarily add to clinician workloads.

We obtained closed-form 95% intervals for the c-statistic and flexible calibration curves using the CalibrationCurves R package.^33^ Flexible calibration curves were drawn using LOcally Estimated Scatterplot Smoothing, which are recommended due to their ability to detect non-linearity in the calibration curve.^34^

Statistical performance measures fail to consider the impact of the decisions made based on predicted probabilities. Clinicians may wait until the patient is severely or terminally ill to initiate end-of-life care discussions, which may not be beneficial for the patient.^35^ A clinician with a large caseload or strong belief in the patient’s chances of recovery may opt to begin discussions only when the estimated risk of death exceeds 40%; a clinician comfortable with initiating end-of-life discussions may prefer a lower threshold of 15%. This trade-off is explored by decision curve analysis, which defines net benefit as the sum of the true positives minus false positives, divided by the sample size and multiplied by the odds of the outcome.^12^ We calculated net benefit using the dcurves R package.^36^

### Post hoc analysis

In both prior research and our study, we observed that the Clinical Frailty Scale demonstrates a non-linear relationship with mortality. As this was noted only after the analysis plan was agreed, we conducted a post hoc analysis in which the Scale was modelled with a spline term, rather than discarding information by using thresholds.^37^ The number of knots for the spline was chosen using the Akaike information criterion.

## Results

Our dataset, described in table 1, contained 4639 patients from 3 major metropolitan hospitals in Queensland, Australia. Prior to missing data imputation, there were just 26 (0.6%) missing Clinical Frailty Scales; all were singly imputed. Survival curves, stratified by Clinical Frailty Scale, are shown in figure 1. The curves show a strong separation by Clinical Frailty Scale with a high risk of death for the highest scores (just 22% surviving at 90 days) and low risk of death for the lowest scores (no deaths at 90 days), although the sample sizes are small in these two extremes (under 10 patients). Survival curves were generally ordered by score, although the gaps between the curves were not constant, indicating a likely non-linear association.

**Table 1:** Patient demographics across hospitals

	Hospital 1	Hospital 2	Hospital 3	Total
Patients	1920	647	2072	4639
Mean age (SD)	84 (5.7)	84 (5.8)	85 (5.9)	84 (5.8)
Female (%)	967 (50)	330 (51)	1191 (57)	2488 (54)
Mean CFS (SD)	4.8 (1.2)	5.2 (1.1)	5.2 (1.3)	5.1 (1.3)
Died within 90 days (%)	447 (23)	113 (18)	396 (19)	956 (21)

CFS, Clinical Frailty Scale.

All model equations are listed in the online supplemental file 1. The tested models are summarised as follows:

- Model 1: 90-day mortality=Clinical Frailty Scale (linear).
- Model 2: 90-day mortality=Clinical Frailty Scale (linear)+age (linear).
- Model 3: 90-day mortality=Clinical Frailty Scale (linear)+age (non-linear).
- Model 4: 90-day Mortality=Clinical Frailty Scale (non-linear)+age (linear).

**Figure 2:**
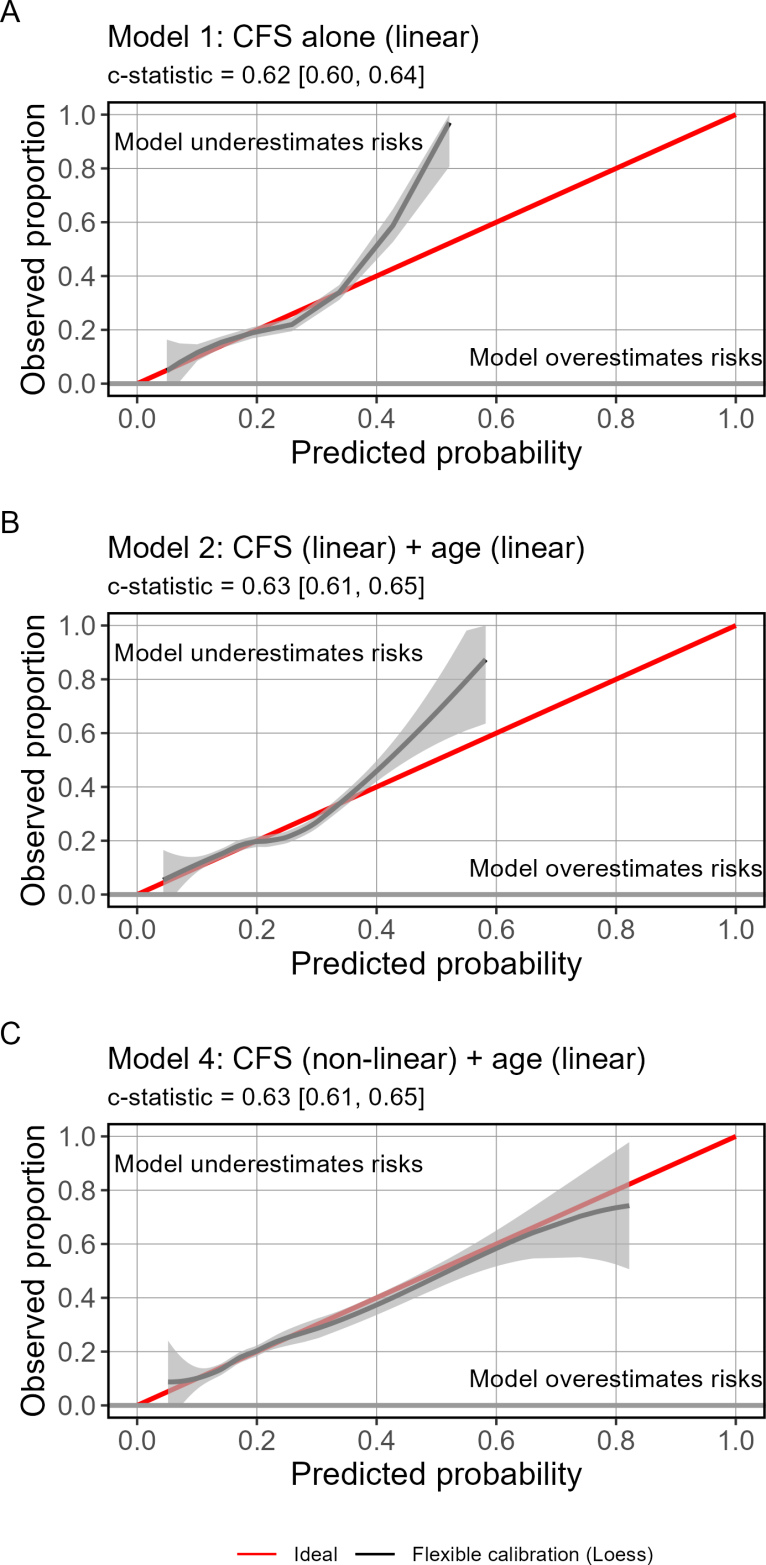
Evaluation of predictive performance for models 1, 2 and 4 to predict 90-day mortality. Model 3 was excluded as a non-linear term for age was not justified. The flexible calibration curve shows significant risk underestimation after predicted probabilities of 0.34 on the x-axis until the Clinical Frailty Scale (CFS) is modelled with a spline term. The grey area is a 95% interval for the curve. The alignment of the black calibration line with the red ideal calibration line for the bottom panel shows a well-calibrated model for predicting in-hospital mortality.

The Clinical Frailty Scale alone (model 1) showed a c-statistic of 0.62 (95% CI 0.60 to 0.64). Calibration was reasonable until predicted probabilities exceeded 34%, or a Clinical Frailty Scale of 7, after which the Clinical Frailty Scale severely underestimated mortality risk (figure 2A).

The likelihood ratio χ² statistic for adding age to the model was 11.7 (p<0.001), suggesting that the linear term for age improved model fit (figure 2B). However, this did little to improve model performance, with a c-statistic of 0.63 (95% CI 0.61 to 0.65) and only slightly improved calibration. A non-linear spline term for age (model 3) was not justified based on a Wald test comparison to a linear association (p=0.84).

**Figure 3:**
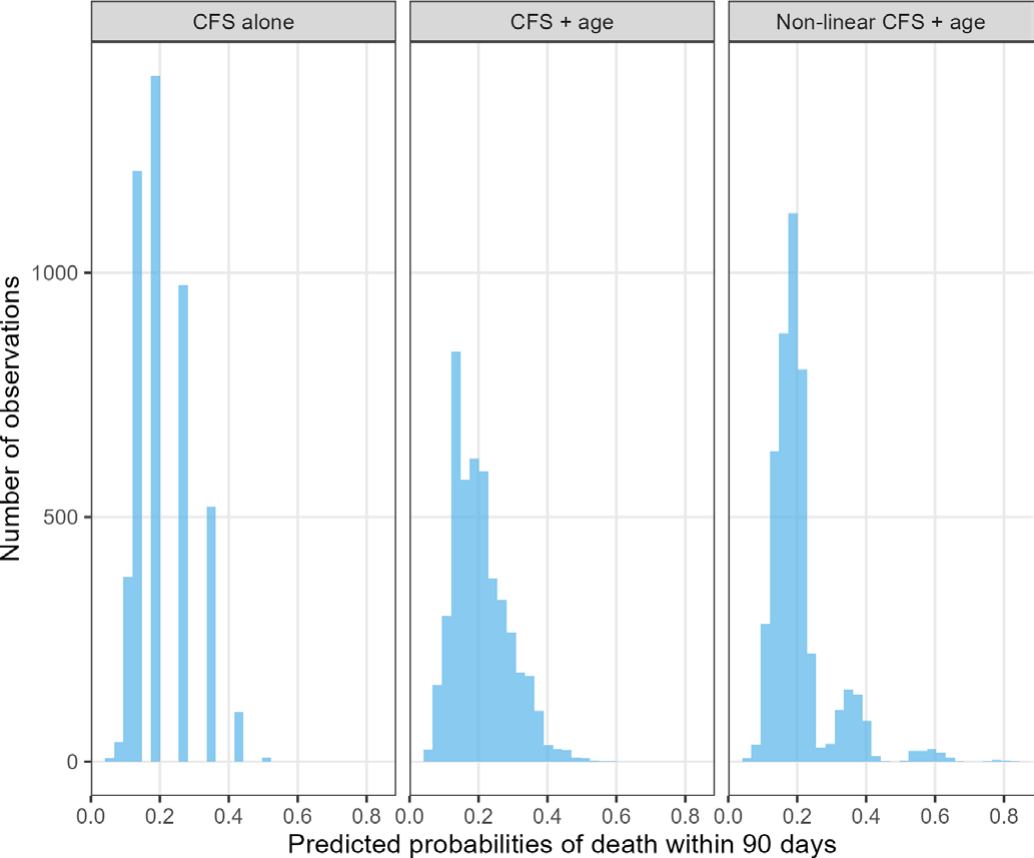
Distribution of predicted death probabilities for the Clinical Frailty Scale (CFS) (linear) model, the CFS (linear)+age (linear) model and the CFS (non-linear)+age (linear) model (n=4639). There is a slightly multimodal risk distribution visible in the predictions made by the last model.

The Wald test showed strong evidence of non-linearity for the Clinical Frailty Scale when fitted with a restricted cubic spline with five knots (p<0.001). The non-linear Clinical Frailty Scale plus age showed improved model fit, with a likelihood ratio χ² statistic of 29.8 (p<0.001). While this improvement did not translate to better discrimination (c-statistic=0.63 (95% CI 0.61 to 0.65)), it did lead to a reasonably well-calibrated model (figure 2C). The addition of age increased the range of possible scores, while the addition of age and a non-linear term for Clinical Frailty Scale showed some multimodality (figure 3). This suggests that there may be distinct phenotypes of patients that can be identified using the Clinical Frailty Scale.

**Figure 4:**
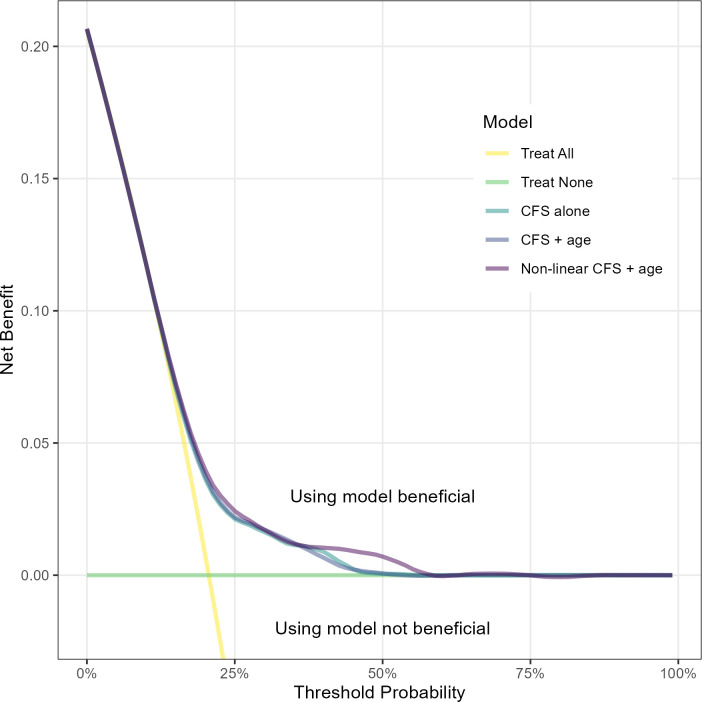
Net benefit plot for each of the three models evaluated. The treat all and treat none lines in this case correspond to having end-of-life care discussions with all and no patients, respectively. Net benefit can be considered a measure of the rate of true positives without increasing the false positive rate as a result of using the model. CFS, Clinical Frailty Scale.

Decision curves (figure 4) showed that the Clinical Frailty Scale could be most useful when the predicted probability was between approximately 20% and 50% probability of death within 90 days. This corresponded primarily to scores of 5–8, inclusive. Coefficients of the model were highly stable, showing no detectable variation (online supplemental file 1).

## Discussion

We found clear evidence of an association between the Clinical Frailty Scale and 90-day mortality, but limited utility for individual patient predictions. Our results suggest that the score should therefore not be used to classify patients as high or low risk, though it remains a useful tool for prompting clinicians to consider patient frailty and its relationship with short-term mortality. While model calibration and net benefit were improved by additional predictors and non-linearity, this would detract from the ease of application, no longer requiring simple mental arithmetic but rather a calculator or application. Net benefit calculations indicated that Clinical Frailty Scale utility for decision-making was greatest when predicted risks exceeded 20%, corresponding roughly to a Clinical Frailty Scale of 5 and above.

The low discrimination of the Clinical Frailty Scale can likely be explained by its ordinal values, leading to distinct and relatively homogenous subgroups. The c-statistic is calculated by taking pairs of patients, one who died within 90 days and one who did not, and measuring the proportion of pairs in which the patient who died had a higher predicted risk. Risk distributions with high homogeneity within subgroups can lead to lower c-statistics.^38^

Despite the relatively low discrimination performance of the models tested in this paper, the c-statistic is not sufficient for determining whether a model is clinically useful. Willingness of clinicians to consider, screen for and discuss end of life care is crucial regardless of their uncertainty in the patient’s prognosis. It is likely that the primary utility of the Clinical Frailty Scale is to prompt clinicians to confront this fact.

The non-linear association with mortality for scores of over 7 is potentially useful information for clinicians who use the scale. In our sample, scores of 8 (very severely frail) and 9 (terminally ill) had risks that were not proportional to a linear increase. These two highest categories could be somewhat separated because the description for a score of 7 includes ‘not at high risk of dying’, whereas the descriptor for a score of 8 includes ‘approaching the end of life’. A modified scale could include a new category between 7 and 8 that attempts to halve the risk distance. However, any changes to the scale would need to be balanced against its widespread use and ease of interpretation.

National US guidelines in oncology recommend end-of-life care discussions take place for patients with a predicted life expectancy of under 12 months,^39^ despite several prognostic models for advanced cancer at the time of release of those guidelines reporting a c-statistic of 0.63 or less.^40^ This suggests that rather than basing discussions around the statistical uncertainty of death, frailty measures may prompt clinicians to initiate those discussions sooner rather than when the patient is terminally ill and unable to sufficiently prepare.

### Comparison with prior literature

To our knowledge, our study is the first to thoroughly examine the Clinical Frailty Scale in terms of its prognostic value and benefit towards decision-making. A 2020 review identified four external validation studies of the Clinical Frailty Scale^41^ on older patients in acute medical contexts, predicting mortality within 30 days up to 5 years. The earliest study included, Rockwood *et al*’s development of the Clinical Frailty Scale,^22^ reported a c-statistic of 0.70 for 5-year mortality. This time frame, while long, may be appropriate for improving the awareness and preparedness of older patients and their families for the inevitability of ageing and death. Conversely, a c-statistic of 0.72 (95% CI 0.69 to 0.75) was reported for predicting in-hospital mortality,^42^ which may be too short for end-of-life care discussions.

The Clinical Frailty Scale has been validated in EDs,^43^ with a far lower mortality rate than in our sample. The authors grouped scores into four categories, reporting calibration and the c-statistic of the Clinical Frailty Scale after adjusting for age, sex and condition, reporting a well-calibrated model and a c-statistic of 0.81 (95% CI 0.77 to 0.85). These changes mean that the authors have not externally validated the Clinical Frailty Scale, but instead published a new, updated model containing the Clinical Frailty Scale, as we have done in this paper with models 2, 3 and 4. A similar approach was taken by McIsaac *et al* who adjusted the Clinical Frailty Scale for the type of surgery in postoperative patients to obtain a c-statistic of 0.67.^44^ These findings align with our suggestion that the Clinical Frailty Scale alone may not be sufficiently discriminating for mortality prediction, but can form a valuable component of a larger model. We refer readers interested in the distinction between new and validated models to Moons *et al*.^45^

### Limitations

While the Clinical Frailty Scale was typically calculated by clinical staff on the patient’s admission to hospital, in some cases, it was calculated retrospectively based on clinical notes. Prior research has shown that the Scale can reliably be applied retrospectively.^46^ To ensure that this limitation was mitigated as thoroughly as possible, auditors were asked to find additional documentation that supported the assigned number on the Scale, including the degree of help the patient had at home, the involvement of the family in managing routine daily tasks, and any associated information that could help with correct attribution of the Scale. However, this may exacerbate the subgroup homogeneity problem described above.

Our sample was a relatively unwell group as they had been admitted to hospital and screened as being at risk of non-beneficial care, making them more unwell than the population of older people presenting to the ED. However, this limitation is mitigated by our choice of evaluation metrics. The c-statistic applies over the full range of all predicted values due to its equivalence to the AUC. Additionally, there was still substantial variation in the Clinical Frailty Scale in our sample (figure 2) and the 90-day mortality rate was 21%, indicating that there was a clear need for prognostic tools to differentiate patients at the end of life even among this relatively unwell sample. Clinicians are required to make decisions about end-of-life care after ED presentation, hence having a simple tool that could predict mortality would be of significant benefit, but creating a highly accurate tool may require the addition of many additional parameters given the complex histories of many older patients.

Binary mortality values do not explore the association between scores and the timing of mortality. By splitting our outcome into a simple measure of whether the patient died within 90 days, we are discarding information about the timing of the patient’s death. Our choice was driven primarily by coherence with previously published research and the ease of interpretation of logistic regression. While a survival model for generating mortality predictions was out of scope for this study, it is a powerful alternative for mortality prediction.^47^

## Conclusions

In this external validation of the Clinical Frailty Scale, we examined its utility in identifying older patients in hospital at risk of being at the end of their life. Higher scores were strongly associated with an increased risk of death; however, the scale should not be used as a highly discriminating clinical prediction tool. The scale is relatively simple to complete and may have value in prompting or framing discussions around end of life.

## Supplementary material

10.1136/bmjopen-2025-108419 online supplemental file 1

## Data Availability

Data are available in a public, open access repository.
