# Supplementary material for: Validation of the Clinical Frailty Scale for predicting 90-day mortality in hospitalised older adults screened as at risk of nearing the end of life in Queensland, Australia: a multisite observational study
Source: BMJ Open. 2025 Nov 12;15(11):e108419. doi: 10.1136/bmjopen-2025-108419 (PMC12612731; doi:10.1136/bmjopen-2025-108419)
Supplement: online supplemental file 1 [file bmjopen-15-11-s001.docx]

**Supplementary tables and figures**

**TRIPOD checklist**

| **Section/Topic** | **Item** | **Checklist Item** | **Page** |
| --- | --- | --- | --- |
| **Title and abstract** | | | |
| Title | 1 | Identify the study as developing and/or validating a multivariable prediction model, the target population, and the outcome to be predicted. | 1 |
| Abstract | 2 | Provide a summary of objectives, study design, setting, participants, sample size, predictors, outcome, statistical analysis, results, and conclusions. | 2 |
| **Introduction** | | | |
| Background and objectives | 3a | Explain the medical context (including whether diagnostic or prognostic) and rationale for developing or validating the multivariable prediction model, including references to existing models. | 3 |
|  | 3b | Specify the objectives, including whether the study describes the development or validation of the model or both. | 3-4 |
| **Methods** | | | |
| Source of data | 4a | Describe the study design or source of data (e.g., randomized trial, cohort, or registry data), separately for the development and validation data sets, if applicable. | 4 |
|  | 4b | Specify the key study dates, including start of accrual; end of accrual; and, if applicable, end of follow-up. | 4 |
| Participants | 5a | Specify key elements of the study setting (e.g., primary care, secondary care, general population) including number and location of centres. | 4 |
|  | 5b | Describe eligibility criteria for participants. | 4-5 |
|  | 5c | Give details of treatments received, if relevant. | 4 |
| Outcome | 6a | Clearly define the outcome that is predicted by the prediction model, including how and when assessed. | 5 |
|  | 6b | Report any actions to blind assessment of the outcome to be predicted. | 4 |
| Predictors | 7a | Clearly define all predictors used in developing or validating the multivariable prediction model, including how and when they were measured. | 5-6 |
|  | 7b | Report any actions to blind assessment of predictors for the outcome and other predictors. | 4 |
| Sample size | 8 | Explain how the study size was arrived at. | 6 |
| Missing data | 9 | Describe how missing data were handled (e.g., complete-case analysis, single imputation, multiple imputation) with details of any imputation method. | 5 |
| Statistical analysis methods | 10a | Describe how predictors were handled in the analyses. | 5-6 |
|  | 10b | Specify type of model, all model-building procedures (including any predictor selection), and method for internal validation. | 5-8 |
|  | 10d | Specify all measures used to assess model performance and, if relevant, to compare multiple models. | 6 |
| Risk groups | 11 | Provide details on how risk groups were created, if done. | NA |
| **Results** | | | |
| Participants | 13a | Describe the flow of participants through the study, including the number of participants with and without the outcome and, if applicable, a summary of the follow-up time. A diagram may be helpful. | 8 |
|  | 13b | Describe the characteristics of the participants (basic demographics, clinical features, available predictors), including the number of participants with missing data for predictors and outcome. | 8 |
| Model development | 14a | Specify the number of participants and outcome events in each analysis. | 8 |
|  | 14b | If done, report the unadjusted association between each candidate predictor and outcome. | NA |
| Model specification | 15a | Present the full prediction model to allow predictions for individuals (i.e., all regression coefficients, and model intercept or baseline survival at a given time point). | Sup |
|  | 15b | Explain how to the use the prediction model. | 11 |
| Model performance | 16 | Report performance measures (with CIs) for the prediction model. | 9-11 |
| **Discussion** | | | |
| Limitations | 18 | Discuss any limitations of the study (such as nonrepresentative sample, few events per predictor, missing data). | 13-14 |
| Interpretation | 19b | Give an overall interpretation of the results, considering objectives, limitations, and results from similar studies, and other relevant evidence. | 11-12 |
| Implications | 20 | Discuss the potential clinical use of the model and implications for future research. | 12 |
| **Other information** | | | |
| Supplementary information | 21 | Provide information about the availability of supplementary resources, such as study protocol, Web calculator, and data sets. | 4 |
| Funding | 22 | Give the source of funding and the role of the funders for the present study. | 14 |

**Model equations**

Model equations can be obtained from the R code simply enough using rms::Function(fit). They are shown here for ease of extraction.

| **Model** | **Covariates** | **Equation** |
| --- | --- | --- |
| 1 | CFS (Linear) | -3.3374107 + 0.38067698 * CFS |
| 2 | CFS (Linear) + Age (Linear) | -5.0790319 + 0.36253279 * CFS + 0.021719865 * Age |
| 3 | CFS (Linear) + Age (Non-linear) | -5.3513905 + 0.36265963 * CFS + 0.025144873 * Age - 0.000015393926 * pmax(Age – 76, 0)^3 + 0.000030787853 * pmax(Age – 84, 0)^3 -0.000015393926 * pmax(Age – 92, 0)^3 |
| 4 | CFS (Non-linear) + Age (Linear) | -5.0093516 + 0.35048558 * CFS –  0.010752086 * pmax(CFS – 3, 0)^3 –  0.050818238 * pmax(CFS – 4, 0)^3 +  0.30972694 * pmax(CFS – 5, 0)^3 –  0.42399082 * pmax(CFS – 6, 0)^3 +  0.17583421 * pmax(CFS – 7, 0)^3 +  0.023016197 * Age |

**Model stability**

Calibration plots were created using the internal bootstrap with resampling. The complete lack of any spread for each model type shows that predicted risks are nearly identical across bootstrap iterations. We would expect a fanning effect for each line if there was instability. The lines were created using 80% transparency; the complete lack of transparency or spread visible on the plot indicates that every line overlaps with the others by model type.

**
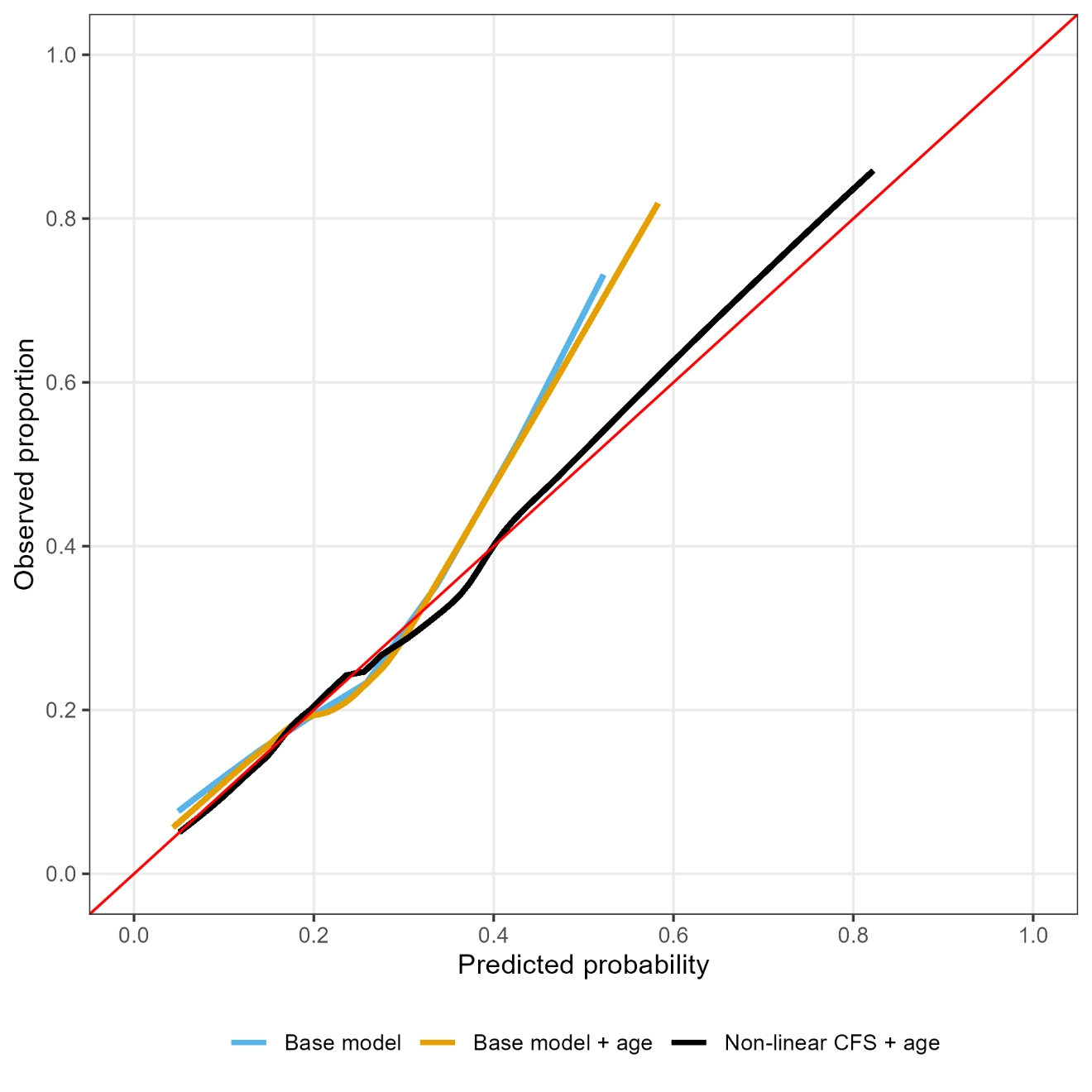
**

*Figure S1: Model stability plot of the calibration curves. There appears to be no instability, indicating that model coefficients are stable across bootstrap iterations.
CFS: Clinical Frailty Scale.*

Similarly, the average prediction error is virtually undetectable. We would expect spread across the y-axis if this were an unstable model. These results across both figures are expected given there were only a few degrees of freedom and our sample was relatively large.


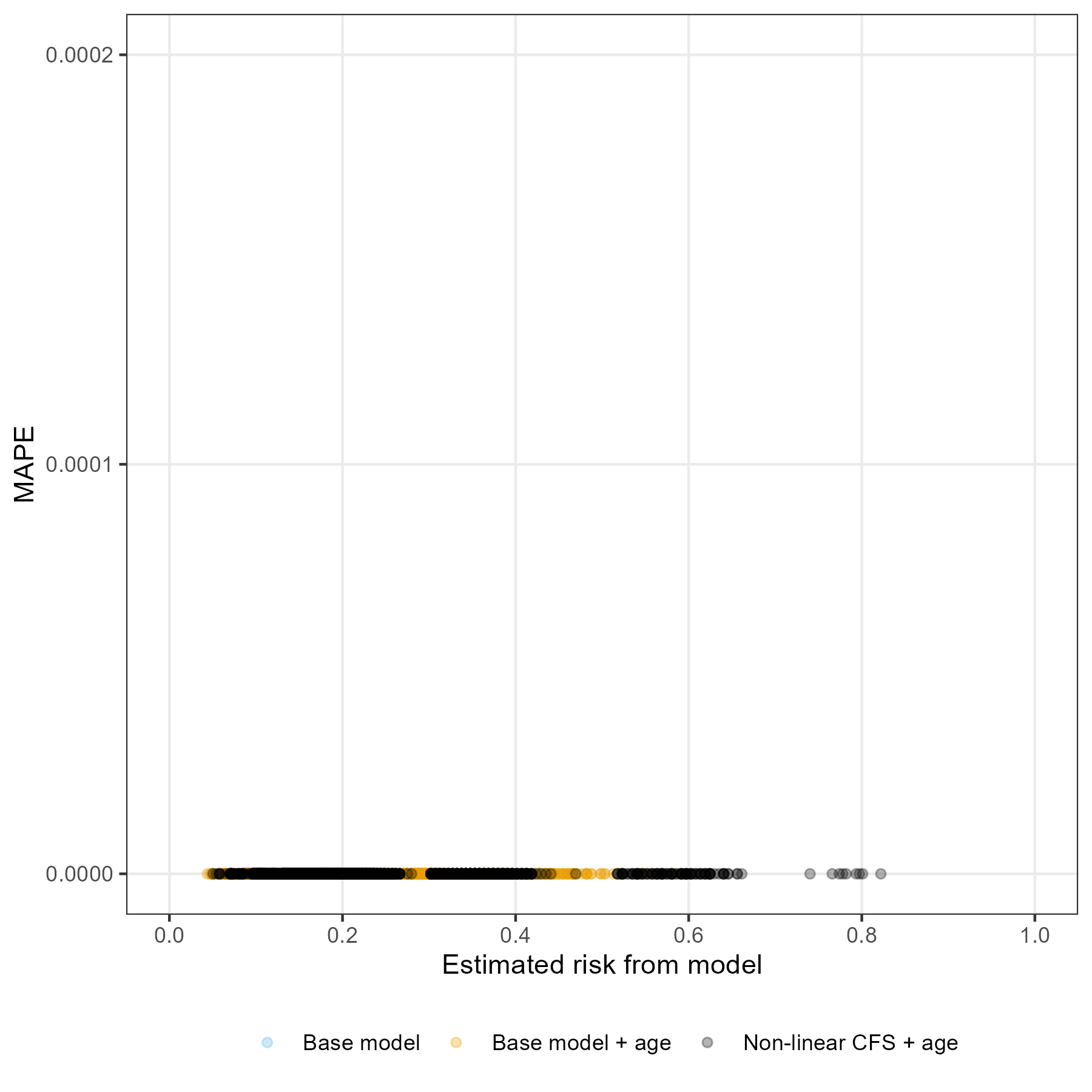


*Figure S2: Model stability plot of the mean average prediction error (MAPE). There appears to be no instability, indicating that model coefficients are stable relative to the predicted outcome.
CFS: Clinical Frailty Scale.*
