# Supplementary material for: Validation of the Clinical Frailty Scale for predicting 90-day mortality in hospitalised older adults screened as at risk of nearing the end of life in Queensland, Australia: a multisite observational study
Source: BMJ Open. 2025 Nov 12;15(11):e108419. doi: 10.1136/bmjopen-2025-108419 (PMC12612731; doi:10.1136/bmjopen-2025-108419)
Supplement: Supplementary file 2 [file bmjopen-2025-108419.reviewer_comments.pdf]

PEER REVIEW HISTORY

BMJ Open publishes all reviews undertaken for accepted manuscripts. Reviewers are asked to complete a checklist review form and are provided with free text boxes to elaborate on their assessment. These free text comments are reproduced below.

ARTICLE DETAILS

Title (Provisional)

Validation of the Clinical Frailty Scale for predicting 90-day mortality in hospitalised older adults screened as at risk of nearing the end of life in Queensland, Australia: A multisite observational study

Authors

Blythe, Robin; Brown, Christine; White, Nicole Marie; Hillman, Ken; Barnett, Adrian Gerard

---

VERSION 1 - REVIEW

**Reviewer**	**1**
**Name**	**Richardson, Alice**
**Affiliation**	**Australian National University, NCEPH**
**Date**	**27-Aug-2025**
**COI**	**None**

In this paper the authors report on a secondary analysis of randomised clinical trial data that provides evidence for the validity of the Clinical Frailty Scale. Validity is assessed in terms of calibration, decision curves and the c-statistics (area under the ROC curve).

The results showed that the Scale alone has a non-linear relationship with mortality, which reduces its clinical benefit somewhat. However, much of this benefit can be regained by including other predictors and/or non-linear terms in modelling the relationship.

I don't have any major comments to make as I think the authors have taken an important topic of clinical relevance, stated clear aims (lines 89 – 98), explained their statistical methods meticulously and provided suitable analysis to back up their conclusions.

That said, I think the third hypothesis leaps into mentioning a "model" without specifying what this model is actually for. Even the model statements in lines 217 – 220 state predictors but not an outcome. The authors should consider a way to link these parts of the paper together with greater clarity.

I noticed that the paper ends with Limitations, and there does not seem to be a Conclusion as such. Within the structure imposed by the journal, the authors should consider closing

with a clear statement of what was done and what has been achieved rather than what remains to be done.

Minor comments

Line 95. I do not like the term “sample population” as usual terminology has the sample as a subset of the population. Does this phrase need to be replaced?.

Line 147. I think it is unclear how the distribution of frailty scores can be assessed using a Kaplan-Meier curve as such curves are usually intended for survival times or times-to-event more generally. Does this sentence need to be expanded or clarified?

Line 417. This reference needs checking.

In conclusion, I am pleased to recommend that the paper be accepted for publication once these matters have been attended to.

**Reviewer**	**2**
**Name**	**Wu, Wenwen**
**Affiliation**	**Hubei University of Medicine, School of health science**
**Date**	**06-Sep-2025**
**COI**	**None**

This manuscript presents an external validation study of the Clinical Frailty Scale (CFS) for predicting 90-day mortality in a large cohort of hospitalized older adults in Australia. The topic is highly relevant to clinical practice in geriatric and palliative care. The major strength of the study is its large sample size and the application of rigorous statistical techniques (c-statistic, calibration curves, decision curve analysis) to evaluate the CFS's performance beyond simple discrimination. While the study is well-motivated and the analysis appears sound, there are several major concerns that need to be addressed before the manuscript can be considered for publication.

Comments:

(1) Title and Abstract Refinement: The population is now clearly defined as inpatients screened positive using CriSTAL or SPICT tools. The title and abstract should be updated to reflect this specificity for accuracy and to manage reader expectations regarding generalizability. For example, the title could be rephrased to: "Validation of the Clinical Frailty Scale for predicting 90-day mortality in hospitalised older adults screened for risk of nearing end of life". This small change significantly enhances the paper's precision.

(2) The finding that the CFS underestimates risk at scores of 7+ is clinically crucial. This should be emphasized in the abstract's conclusion to alert clinicians that a score of 7 or 8 may represent an even higher risk than the raw score implies.

(3) Clarification on "First Study" Claim: The discussion effectively compares the study to prior literature. The claim of being the "first to thoroughly examine" the CFS is supported by the comprehensive suite of metrics used (calibration, decision curves). To strengthen this further, consider slightly tempering the language in the "Strengths and limitations" section to focus on the comprehensiveness of the validation (e.g., "This is among the first studies to evaluate... using a comprehensive assessment of discrimination, calibration, and clinical utility") rather than being the absolute first, which can sometimes be contentious.

(4) Abstract Background: The sentence "We externally validated the Scale..." could be slightly refined to set clearer expectations, e.g., "We conducted an external validation of the Scale to assess its performance for predicting 90-day mortality in a cohort of admitted older patients who had screened positive for being at risk of nearing end-of-life."

(5) Methods - Data Collection: Please provide the prevalence of positive screens for CriSTAL and SPICT in the overall admitted population, if available, to give readers a sense of how selective the inclusion criteria were.

(6) The results mention risk was underestimated without a non-linear transformation. Could you speculate in the discussion why the relationship between CFS and mortality might be non-linear? For instance, is there a "threshold effect" or does the risk accelerate beyond a certain level of frailty?

(7) The discussion on national US oncology guidelines (ref 38-39) is a powerful argument. Was the 90-day timeframe chosen, in part, to be a more conservative and challenging benchmark than the 12-month guideline??

VERSION 1 - AUTHOR RESPONSE

Reviewer: 1
Dr. Alice Richardson, Australian National University
In this paper the authors report on a secondary analysis of randomised clinical trial data that provides evidence for the validity of the Clinical Frailty Scale. Validity is assessed in terms of calibration, decision curves and the c-statistics (area under the ROC curve). The results showed that the Scale alone has a non-linear relationship with mortality, which reduces its clinical benefit somewhat. However, much of this benefit can be regained by including other predictors and/or non-linear terms in modelling the relationship.

I don’t have any major comments to make as I think the authors have taken an important topic of clinical relevance, stated clear aims (lines 89 – 98), explained their statistical methods meticulously and provided suitable analysis to back up their conclusions.

That said, I think the third hypothesis leaps into mentioning a "model" without specifying what this model is actually for. Even the model statements in lines 217 – 220 state predictors but not an outcome. The authors should consider a way to link these parts of the paper together with greater clarity.
Response: We have been more specific about the "model" on line 86 and in the following bullet points. We've added the outcome to the model statements.

I noticed that the paper ends with Limitations, and there does not seem to be a Conclusion as such. Within the structure imposed by the journal, the authors should consider closing with a clear statement of what was done and what has been achieved rather than what remains to be done.
Response: We have now added a conclusion paragraph after the limitations.

Minor comments
Line 95. I do not like the term "sample population" as usual terminology has the sample as a subset of the population. Does this phrase need to be replaced?.
Response: We agree and have just used "sample" (line 95).

Line 147. I think it is unclear how the distribution of frailty scores can be assessed using a Kaplan-Meier curve as such curves are usually intended for survival times or times-to-event more generally. Does this sentence need to be expanded or clarified?
Response: Thanks for flagging this. We have added more justification on how these curves were an exploratory analysis.

Line 417. This reference needs checking.
Response: We have added the volume number for the reference titled "Stability of clinical prediction models developed using statistical or machine learning methods".

In conclusion, I am pleased to recommend that the paper be accepted for publication once these matters have been attended to.
Response: Thanks for your constructive comments.

Reviewer: 2
Prof. Wenwen Wu, Hubei University of Medicine
This manuscript presents an external validation study of the Clinical Frailty Scale (CFS) for predicting 90-day mortality in a large cohort of hospitalized older adults in Australia. The topic is highly relevant to clinical practice in geriatric and palliative care. The major strength of the study is its large sample size and the application of rigorous statistical techniques (c-statistic, calibration curves, decision curve analysis) to evaluate the CFS's performance beyond simple discrimination. While the study is well-motivated and the analysis appears sound, there are several major concerns that need to be addressed before the manuscript can be considered for publication.

Comments:
(1) Title and Abstract Refinement: The population is now clearly defined as inpatients

screened positive using CriSTAL or SPICT tools. The title and abstract should be updated to reflect this specificity for accuracy and to manage reader expectations regarding generalizability. For example, the title could be rephrased to: "Validation of the Clinical Frailty Scale for predicting 90-day mortality in hospitalised older adults screened for risk of nearing end of life". This small change significantly enhances the paper's precision.
Response: We have added "screened as at risk of" to the title and mentioned the screening in the abstract as per your point (4) below.

(2) The finding that the CFS underestimates risk at scores of 7+ is clinically crucial. This should be emphasized in the abstract's conclusion to alert clinicians that a score of 7 or 8 may represent an even higher risk than the raw score implies.
Response: Thanks for this interesting observation, we have added this point to the abstract.

(3) Clarification on "First Study" Claim: The discussion effectively compares the study to prior literature. The claim of being the "first to thoroughly examine" the CFS is supported by the comprehensive suite of metrics used (calibration, decision curves). To strengthen this further, consider slightly tempering the language in the "Strengths and limitations" section to focus on the comprehensiveness of the validation (e.g., "This is among the first studies to evaluate... using a comprehensive assessment of discrimination, calibration, and clinical utility") rather than being the absolute first, which can sometimes be contentious.
Response: The editor requested that we remove this bullet as the strengths and limitations bullets must focus on methods rather than novelty.

(4) Abstract Background: The sentence "We externally validated the Scale..." could be slightly refined to set clearer expectations, e.g., "We conducted an external validation of the Scale to assess its performance for predicting 90-day mortality in a cohort of admitted older patients who had screened positive for being at risk of nearing end-of-life."
Response: We have updated the wording as suggested.

(5) Methods - Data Collection: Please provide the prevalence of positive screens for CriSTAL and SPICT in the overall admitted population, if available, to give readers a sense of how selective the inclusion criteria were.
Response: We agree that this is useful and have added this to the data collection section.

(6) The results mention risk was underestimated without a non-linear transformation. Could you speculate in the discussion why the relationship between CFS and mortality might be non-linear? For instance, is there a "threshold effect" or does the risk accelerate beyond a certain level of frailty?
Response: Thanks for raising this interesting point. With a scale from 1 to 9 using qualitative categories, it would be surprising if the association with mortality was linear as this could only happen if every increase in scale had the same sized increase in risk.

The Kaplan-Meier plot indicated that the gaps in risk were higher for scores 8 and 9. We have added a paragraph to the discussion.

(7) The discussion on national US oncology guidelines (ref 38-39) is a powerful argument. Was the 90-day timeframe chosen, in part, to be a more conservative and challenging benchmark than the 12-month guideline??
Response: The main reason for using a 90-day timeframe was the feeling that this would be the most clinically useful timeframe. Patients in this group can often be re-admitted to hospital within a few months and unnecessary readmissions were one of the negative outcomes that the trial aimed to reduce.

VERSION 2 - REVIEW

**Reviewer**	**1**
**Name**	**Richardson, Alice**
**Affiliation**	**Australian National University, NCEPH**
**Date**	**02-Oct-2025**
**COI**	

In the revised paper the authors have addressed all the issues that I raised in my report in a satisfactory manner. I am pleased to recommend that the paper now be accepted for publication.
